# Beyond immune suppression: regenerative programs in regulatory T cells

**DOI:** 10.1016/j.cellin.2026.100355

**Published:** 2026-08-24

**Authors:** Yannan You, Xianting Hu, Yueqing Xue, Zhi Liu

**Affiliations:** a Shanghai Immune Therapy Institute, Renji Hospital, Shanghai Jiao Tong University School of Medicine, Shanghai, China; b Department of Otolaryngology, Head and Neck Surgery, Eye and ENT Hospital, Fudan University, Shanghai, China

**Keywords:** Regulatory T cells, Tissue repair, Immune suppression, Regenerative immunology

## Abstract

Regulatory T cells (Tregs) are traditionally recognized for their indispensable roles in maintaining immune tolerance and suppressing excessive inflammation. However, accumulating evidence has revealed an unexpected regenerative dimension of Treg biology across multiple tissues. Here, regenerative function refers to the capacity of Tregs to directly regulate stem cells, stromal cells, epithelial cells, and other non-immune tissue cells through specialized reparative mediators, thereby contributing to restoration of tissue structure, cellular composition, or function. Emerging studies demonstrate that these regenerative activities are triggered by tissue injury and coordinated by diverse reparative signals. Notably, in selected experimental settings, specific tissue-regenerative pathways can be mechanistically distinguished from canonical immune-regulatory functions of Tregs, although these programs frequently operate in a coordinated and complementary manner during physiological tissue restoration. In this review, we summarize tissue-adapted regenerative programs employed by Tregs across diverse organs, discuss common principles underlying regenerative Treg biology, and evaluate evidence for dedicated regenerative Treg states. Finally, we propose a conceptual framework in which Tregs function as dual-purpose regulators of both immune homeostasis and tissue restoration, highlighting their broader significance in physiology, disease, and regenerative medicine.

## 1 Introduction

Foxp3^+^ Tregs are a subset of CD4^+^ T cells that specialize in maintaining immune tolerance and suppressing aberrant immune responses (Sakaguchi et al., 2008, 2020). Genetic ablation or functional deficiency of Tregs, as exemplified by Foxp3 mutations in scurfy mice or in human patients with immune dysregulation, polyendocrinopathy, enteropathy, X-linked (IPEX) syndrome, results in fatal inflammation and tissue damage (Josefowicz et al., 2012; Rudensky, 2011). Tregs can be broadly categorized according to their developmental origin: thymic-derived Treg (tTreg) cells arise as a distinct lineage during thymic selection, whereas peripherally induced Treg (pTreg) cells are generated from naïve CD4^+^ T cells in peripheral tissues such as the colon (Tanoue et al., 2016). Following their maturation, Tregs emigrate from the thymus and seed peripheral compartments, where they exist both as a circulating pool within secondary lymphoid organs (e.g., spleen, lymph nodes) and as tissue-resident populations that adapt to and persist within virtually all non-lymphoid tissues. These context-dependent adaptations endow Tregs with specialized functions in maintaining local immune homeostasis and tissue integrity (Munoz-Rojas et al., 2021) (Fig. 1).

**Fig. 1:**
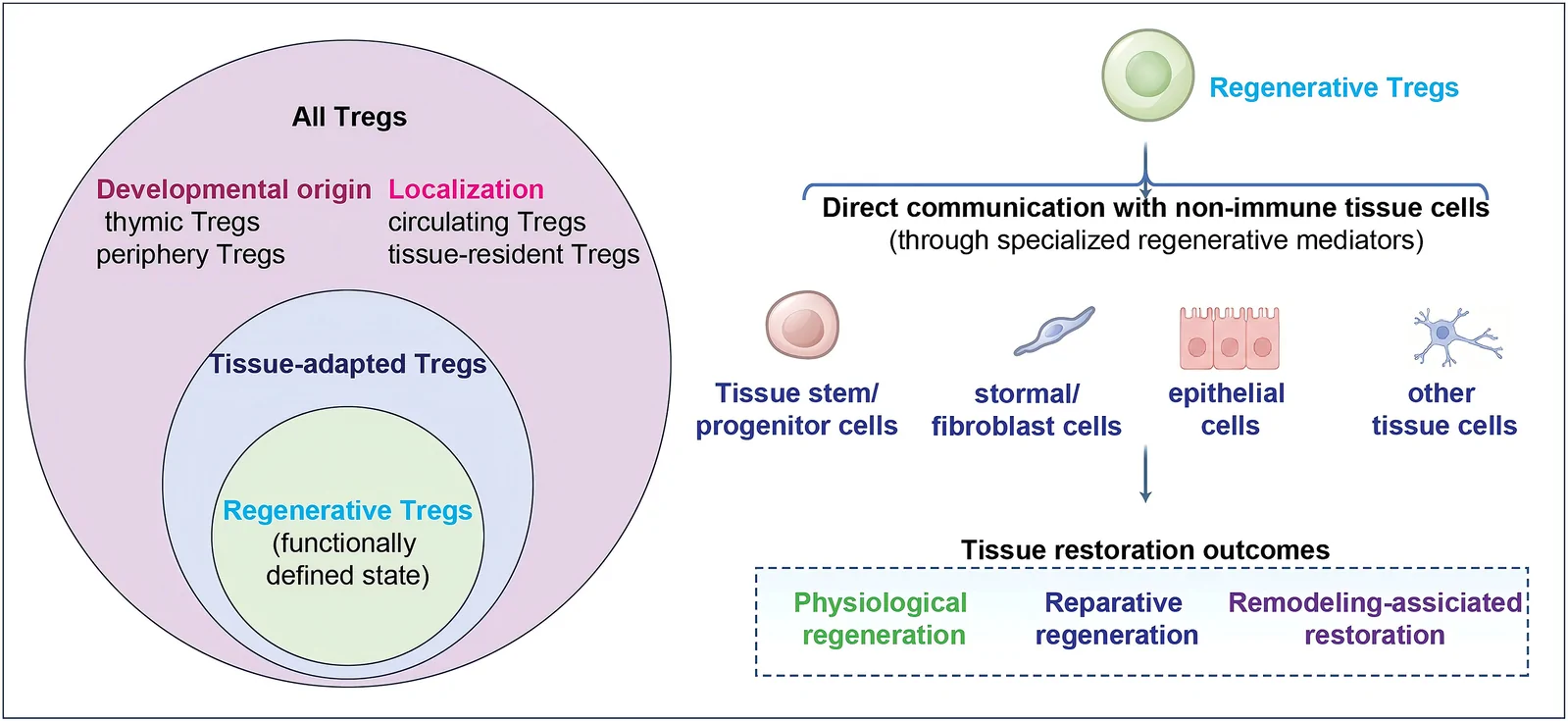
**Conceptual definition of regenerative Tregs as a functional state.** Regenerative Tregs are defined by acquisition of a tissue-regenerative program rather than by developmental origin, tissue localization, or expression of individual regenerative molecules. Both tTregs and pTregs, as well as circulating and tissue-resident Tregs, can acquire regenerative capacity in response to local environmental cues. Through direct communication with non-immune tissue cells, including stem/progenitor cells, stromal cells, epithelial cells, and other parenchymal populations, regenerative Tregs contribute to diverse tissue restoration outcomes, including physiological regeneration, reparative regeneration, and remodeling-associated restoration. This functional state represents an additional modality of Treg biology that complements their canonical immune-regulatory activity.

Since the identification of Foxp3 as the master regulator of Tregs in 2003, extensive research has revealed their inherent capacity to suppress excessive immune responses through production of IL-10, transforming growth factor-beta 1 (TGF-β1), IL-35; sequestration of IL-2 through surface CD25; expression of surface protein CTLA-4; and metabolic disruption (Josefowicz et al., 2012; Sakaguchi et al., 2008). Over the past 15 years, however, accumulating evidence has revealed that Tregs also actively regulate regenerative processes across diverse organs, including skeletal muscle, skin, lung, central nervous system, heart, bone, tendon, and oral mucosa (Ali et al., 2017; Arpaia et al., 2015; Arvind et al., 2025; Burzyn et al., 2013; Chen et al., 2024, 2026; Ito et al., 2019; Li et al., 2019; Liu et al., 2022; Xia et al., 2020). In this review, we use the term regeneration broadly to encompass both physiological regeneration, which maintains or renews tissues under homeostatic conditions, and reparative regeneration, which restores tissue structure, cellular composition, or function following injury. Depending on the intrinsic regenerative capacity and biological context of individual tissues, activation of Treg regenerative programs may contribute to cellular replacement, remodeling, or functional recovery. In these settings, Tregs have been shown to directly modulate non-immune tissue cells—such as stem cells, stromal cells, and epithelial cells—through specialized regenerative mediators, thereby promoting tissue restoration (Loffredo et al., 2024; Sharma et al., 2018).

These findings raise an important conceptual question: how do Tregs integrate immune regulation with tissue regeneration? We propose that Tregs operate through two complementary functional programs: an immune-regulatory program that represents their canonical role in controlling immune responses, and a tissue-regenerative program that enables direct regulation of non-immune tissue cells through specialized reparative signals and mediators. Regenerative Tregs refer to Tregs in which the tissue-regenerative program is engaged, allowing them to coordinate tissue restoration while retaining their conserved immune-regulatory functions. The biological outcomes of these programs vary according to tissue context, ranging from physiological regeneration and reparative regeneration to remodeling-associated restoration. Importantly, these two programs are conceptually distinguishable but functionally interconnected, with their relative contribution varying according to tissue context, injury type, and disease state. Regenerative capacity is therefore not determined solely by tissue residency, developmental origin, or expression of individual molecules, but by acquisition of a tissue-regenerative program in response to local environmental cues. Thus, regenerative Treg states can emerge from either resident or recruited Tregs following adaptation to tissue-specific physiological or injury-associated signals, and represent an additional functional modality that complements their conserved immune-regulatory functions.

Although immune regulation remains an essential component of Treg-mediated tissue restoration, the mechanisms through which Tregs communicate with non-immune tissue cells and activate regenerative programs are less well understood. Therefore, rather than revisiting their established immunosuppressive roles in autoimmunity, allergy, and cancer, which have been extensively reviewed elsewhere (Dominguez-Villar et al., 2018; Imianowski et al., 2025; Noval Rivas et al., 2016), we focus here on the molecular signals, cellular interactions, and therapeutic potential of regenerative Treg programs.

## 2 From immune regulation to tissue restoration: the emergence of regenerative Treg biology

The outset of Treg research can be traced back to the early 1970s when studies reported that 3-day postnatal thymectomy could induce multiorgan autoimmune diseases, indicating suppressor T cells (now referred called Tregs) exist in the thymus (Sakaguchi et al., 2007). In the following 3 decades, although several groups reported that cell surface proteins such as CD5^hi^, CD45RB^low^, and CD25^+^ can be used to distinguish suppressor T cells from other inflammatory/pathogenic T cells, the skepticism of suppressor T cells remains unresolved due to lack of specificity of these markers (Rudensky, 2011). The breakthrough that led to the general acceptance of Tregs as a distinct lineage was the identification of Foxp3 as the master regulator of Tregs in 2003 (Fontenot et al., 2003; Hori et al., 2003; Khattri et al., 2003).

Initially identified as a specialized T cell subset in suppressing exuberant immune responses, the early studies primarily centered on Tregs in the lymphoid organs. This perspective shifted in 2009, when a seminal study by the Diane Mathis group revealed that Tregs in visceral adipose tissue (VAT) could regulate insulin sensitivity, putting forward the notion that non-lymphoid tissues harbor unique populations of Tregs that adapt to survive and operate in their home tissue and perform tissue-specialized functions beyond immune suppression (Feuerer et al., 2009). Subsequently, tissue Tregs with unique phenotypes (i.e., transcriptome and T cell receptor (TCR) repertoire) have been identified in multiple tissues, including skeletal muscle, skin, colonic lamina propria, cardiac muscle, lung, liver, and CNS (Munoz-Rojas et al., 2021).

Amid such growing interest in exploring tissue-specialized function of Tregs, the regenerative function of Tregs was first discovered in the skeletal muscle in 2013, also by the Diane Mathis group (Burzyn et al., 2013). They discovered that Tregs, albeit infrequent in the skeletal muscle under steady state, rapidly increased after injury induced by cardiotoxin injection or mild freezing. Transcriptomic and TCR sequencing analysis showed that muscle Tregs display a unique feature compared to their counterparts in the lymphoid tissues and other non-lymphoid tissues. Furthermore, punctual depletion of Tregs in Foxp3-DTR mice during the repair process prolonged the proinflammatory infiltrate and impaired muscle repair and regeneration. While not very surprising that Treg cell depletion results in heightened inflammation, it is quite intriguing that skeletal muscle Tregs produce amphiregulin (Areg), a growth factor belonging to the epithelial growth factor (EGF) family and signals through the EGF receptor, which directly acts on muscle satellite cells to improve muscle regeneration (Burzyn et al., 2013; Kuswanto et al., 2016). Since this initial discovery, the number of tissues in which Tregs exert regenerative functions has continued to grow (Fig. 2).

**Fig. 2:**
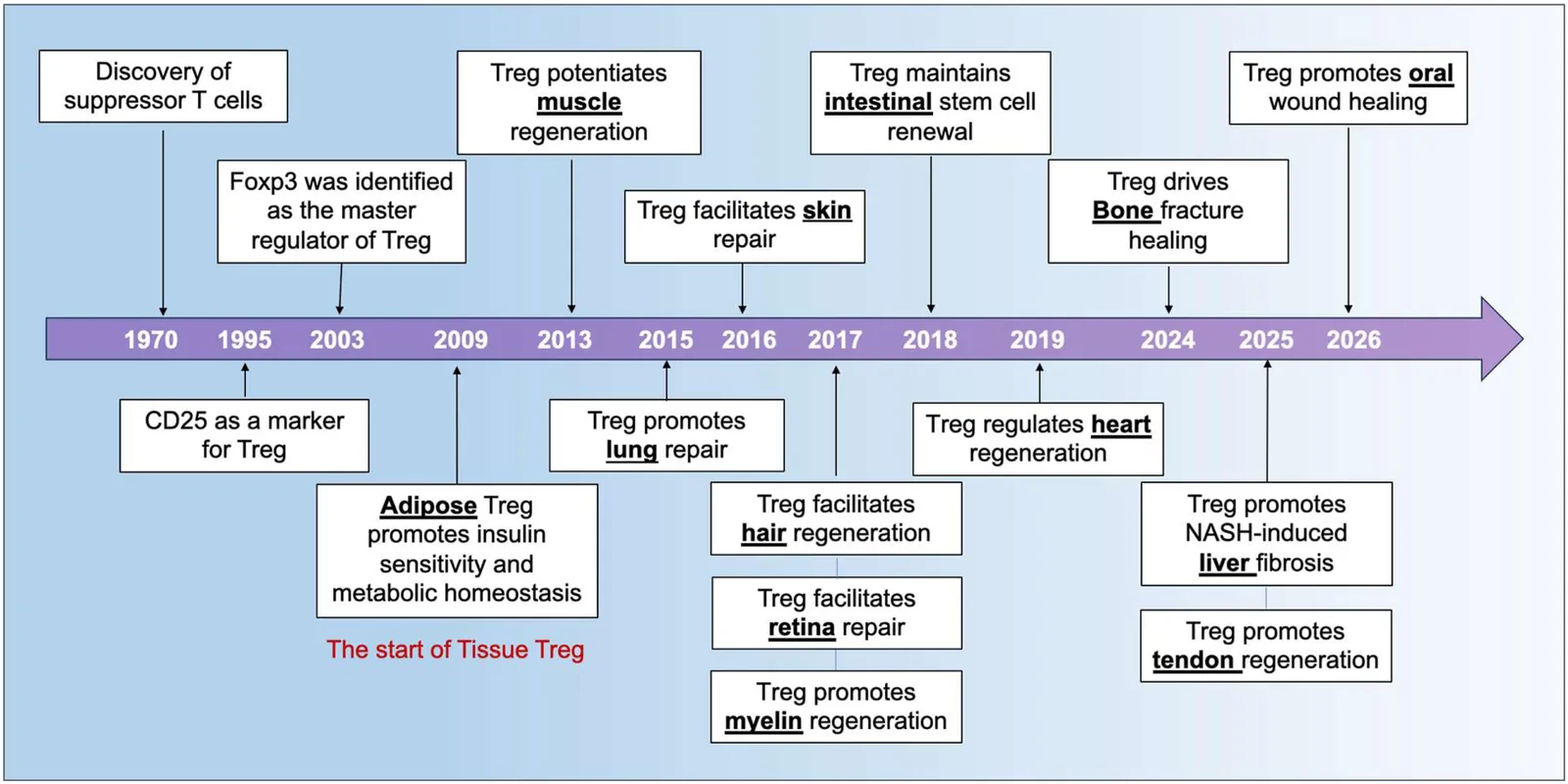
**The discovery of tissue-regenerative functions of Tregs.** Timeline highlighting key discoveries that shaped the field of regenerative Treg biology. Following the identification of Foxp3 as the master regulator of Treg lineage identity, studies initially focused on their immunosuppressive functions in maintaining immune tolerance. Subsequent work demonstrated that Tregs participate in tissue regeneration and restoration in skeletal muscle, skin, lung, thymus, central nervous system, heart, and other organs. The identification of tissue-regenerative mediators and direct Treg–parenchymal cell interactions has expanded the traditional view of Tregs from immune suppressors to multifunctional regulators of both immune homeostasis and tissue restoration.

## 3 Integration of immune-regulatory and tissue-regenerative programs of Tregs during tissue regeneration

Tissues and appendages are complex structures composed of not only different types of tissue cell populations, including tissue stem cells, epithelial cells, endothelial cells, mesenchymal cells, fibroblasts, nerves, but also armed with sophisticated resident and recirculating innate and adaptive immune cells that monitor their health and integrity. In mammals, most tissues are quiescent at steady state. Notable exceptions are the bone marrow, epidermis, hair follicles, and intestine, which undergo physiological regeneration. In quiescent tissues, repair following injury is facilitated by facultative stem/progenitor cells. These cells can be activated to proliferate and differentiate, regenerating damaged tissue through a process known as reparative regeneration (Forbes et al., 2014; Goldman et al., 2020). A large body of evidence shows that successful regeneration requires highly orchestrated actions of immune cells, tissue stem cells, and other parenchymal cells within tissue microenvironments (Naik et al., 2018).

Tissue restoration after injury, often referred to as reparative regeneration, is a highly coordinated process involving sequential but overlapping phases. Immediately after injury, pro-inflammatory cells are recruited to the site of injury for phagocytosis of necrotic tissues and host defense. This acute inflammation phase upon injury not only represents the first line of defense against pathogens, but also is critical to drive tissue reparative regeneration. Nevertheless, a sustained uncontrolled inflammation leads to impaired healing and tissue remodeling. Subsequently, the pro-inflammatory response is dampened via immune cells such as anti-inflammatory macrophages and Tregs, while immune cells also directly participate in stimulating angiogenesis, myofibroblast activation, and tissue stem/progenitor cell proliferation. As inflammation resolves, immune cells are reduced or redistributed, allowing tissue remodeling to restoration of homeostasis (Boothby et al., 2020; Li et al., 2018a).

Within this regenerative process, Tregs serve as important coordinators by integrating immune regulation with tissue restoration. Following injury, Tregs typically accumulate at the transition from the inflammatory to reparative phase, and their depletion disrupts this transition, resulting in impaired tissue regeneration. Consistent with their temporal dynamics, Tregs contribute to regeneration through two complementary functional programs. First, through their immune-regulatory program, Tregs restrain excessive inflammation and establish a permissive immune environment for tissue repair (Munoz-Rojas et al., 2021). Second, through engagement of a tissue-regenerative program, Tregs directly communicate with tissue stem cells and other parenchymal populations through specialized regenerative mediators to promote tissue restoration (Loffredo et al., 2024). Thus, successful regeneration requires coordinated execution of both immune-regulatory and tissue-regenerative programs rather than immune suppression alone (Fig. 3).

**Fig. 3:**
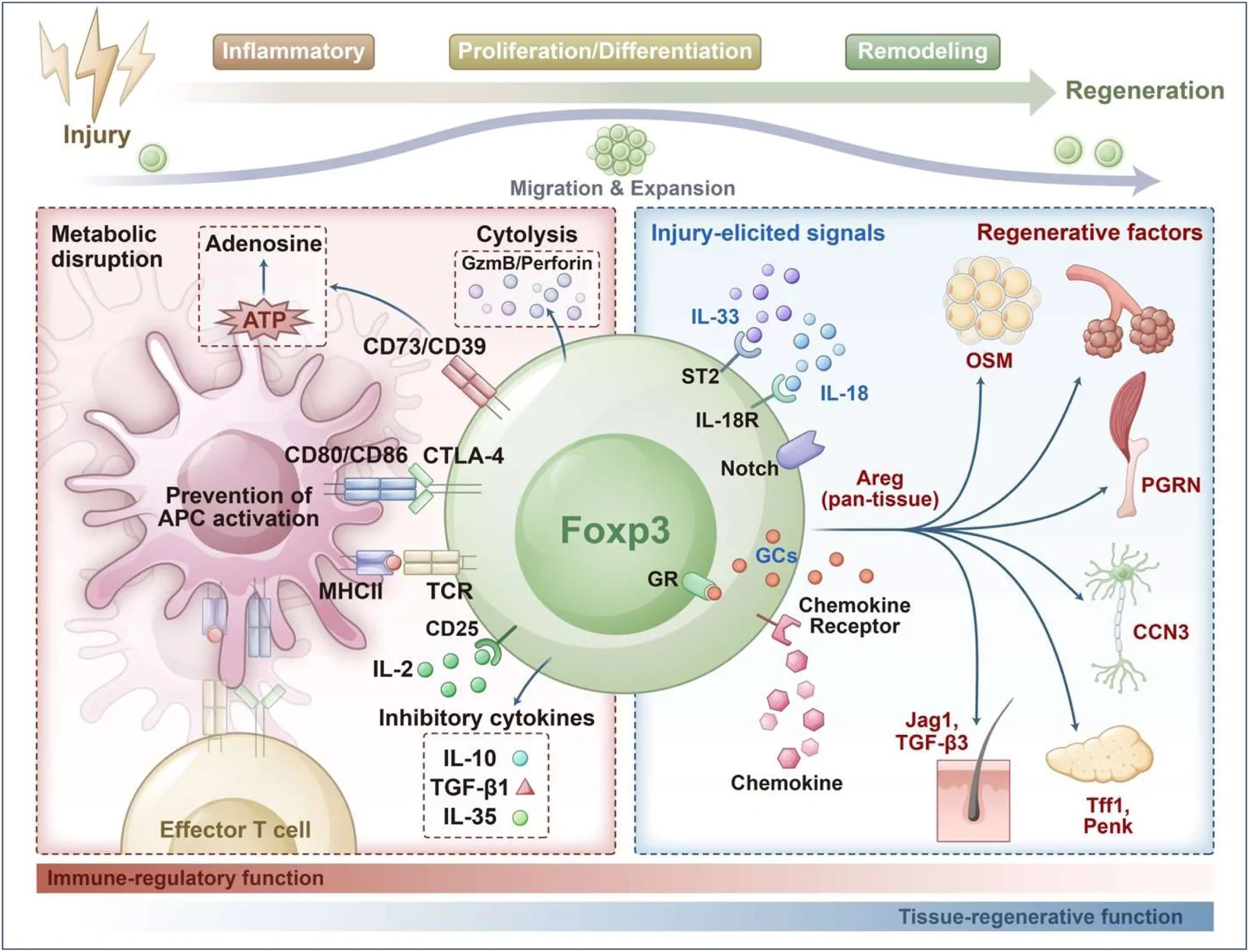
**Overview of the regulatory and regenerative functions of Tregs.** Tregs influx and expand at injured tissues, and reach their peaks at the transition from the inflammatory phase to the proliferation phase. During the inflammatory phase, Tregs exert their canonical immune regulatory function by sequestering IL-2, producing the anti-inflammatory cytokines TGF-β, IL-10, and IL-35, preventing antigen-presenting cell (APC) activation through CTLA-4, disrupting metabolism through CD73 and CD39, and mediating cytolysis through granzyme B and perforin. In the later proliferation stage, Tregs, beyond immunosuppressive roles, can also respond to injury-elicited signals such as IL-18, IL-6, IL-33, glucocorticoids (GCs) and others, produce conserved regenerative factors like Areg, and tissue-specific ones like TGF-β3, Jag1, OSM, PGRN to act on tissue stem cells across multiple tissues to facilitate physiological regeneration, reparative regeneration, and restoration of tissue function.

## 4 Tissue-adapted regenerative programs of Tregs

Across diverse tissues, Tregs have emerged as important regulators of regeneration through coordinated immune-regulatory and tissue-regenerative programs. Importantly, the regenerative outcomes mediated by Tregs vary according to tissue context, including physiological regeneration, reparative regeneration, and restoration of tissue function after injury. Following tissue injury, Tregs accumulate within damaged sites and interact with tissue stem/progenitor cells, stromal cells, epithelial cells, and other parenchymal populations through specialized mediators. Although the specific cellular targets and molecular factors vary among organs, accumulating evidence reveals recurring themes, including injury-induced Treg recruitment, adaptation to local tissue environments, and production of regenerative signals. In the following sections, we summarize representative examples of Treg-mediated tissue restoration in skeletal muscle, lung, skin, thymus, and additional tissues, highlighting both conserved features and tissue-adapted mechanisms that contribute to tissue homeostasis, repair, and regeneration.

### 4.1 Tregs in reparative skeletal muscle regeneration

Skeletal muscle, constituting about 40% of human body weight and 50%-70% of its proteins, is essential for movement, posture, thermoregulation, nutrient storage, and joint stabilization (Frontera et al., 2015). Due to its large size and superficial location, skeletal muscle is vulnerable to frequent injury. Fortunately, it possesses robust regenerative capacity not only in healthy individuals after acute trauma, but also in the context of diseases like muscular dystrophies. Damage to skeletal muscle initiates a highly orchestrated and stereotyped reparative regeneration program regardless of the cause of muscle damage. Muscle regeneration is driven largely by satellite cells, a pool of quiescent muscle stem cells (MuSCs) closely associated with muscle fibers (Tabebordbar et al., 2013). In response to injury, these MuSCs become activated, proliferate, differentiate, migrate, and fuse to form new myofibers. With muscular dystrophies, in which chronic myofiber loss occurs due to genetic defects, the MuSC pool is called on repeatedly, leading to their exhaustion or loss of function over time, dampening the repair process (Relaix et al., 2021).

A very small population of Foxp3^+^ Tregs resides in the skeletal muscle at steady state, but can be rapidly accumulated in the skeletal muscle after various insults, including mild cryoinjury and damage induced by injection of cardiotoxin (CTX), reaching up to 60% of the CD4^+^ T cell compartment (Burzyn et al., 2013; Villalta et al., 2014). This rise coincides with the switch in macrophage phenotype from pro-to anti-inflammatory. In addition to acute injury, Tregs are also enriched in chronic injury of the mdx or Dysf knockout models. Punctual depletion of Foxp3^+^ Tregs after acute muscle injury or in the mdx mouse model of Duchenne muscular dystrophy leads to overproduction of interferon-γ (IFN-γ) by NK and effector T cells, which leads to the failure of transition from proinflammatory M1 macrophage to pro-regenerative M2 macrophage (Panduro et al., 2018; Villalta et al., 2014). Moreover, recent studies also showed that healthful activities of exercise can induce infiltrating inflammatory myeloid cells, and the expansion of Tregs in skeletal muscles, a similar response to injury. In the absence of Tregs, such performance-enhancing effects of exercise training were dampened due to overexuberant production of IFN-γ, muscle mitochondria aberrance (Langston et al., 2023, 2024).

***Signals promote Treg expansion and regenerative phenotype in muscle*.** Muscle Tregs, like other tissue Tregs, highly express ST2 (encoded by *Ilrl1*), a receptor for IL-33, which is an endogenous danger signal that responds to tissue injury. In mice, the accumulation of Tregs depends on IL-33 produced by local mesenchymal stromal cells(mSCs)/Fibro/adipogenic progenitors (FAPs) (Wang et al., 2020). Interestingly, IL-33^+^ mSCs are in proximity to muscle sensory nerves and express the receptor for the calcitonin-gene-related peptide (CGRP), whose up- or down-turn augments or diminishes IL-33 production by mSCs, respectively (Wang et al., 2020). Therefore, the IL-33/ST2 axis is critical for massive Treg accumulation in skeletal muscle after injury. Additionally, although the TCR repertoire of Tregs in muscle is distinct from those in the spleen, a small fraction of Areg^+^ Tregs in the spleen shares TCR sequences with Areg^+^ Tregs or Areg^−^ Tregs in muscle. A transgenic mouse line carrying rearranged *Tcra* and *Tcrb* genes from a Treg clone expanded within muscle after injury displayed a TCR repertoire skewed for the transgene-encoded specificity and an amplified population of muscle Tregs, proving that the tissue accumulation, phenotype acquisition, and functional activities of muscle Tregs were critically dependent on TCR specificity and TCR signaling (Cho et al., 2019).

Recent studies have also shown that exercise rapidly induces expansion of the muscle Treg compartment, and IL-6/IL-6 receptor alpha(IL-6Rα) axis is important for Tregs to control muscle function and regeneration both under endurance exercise and sterile injury by intramuscular injection of glycerol. Exercise promotes a stable induction of muscle-residing Tregs with increased expression of Areg, EGFR, and ST2. Such an effect is dependent on IL-6Rα expression on Tregs, as its deficiency leads to downregulation of EGFR, Areg, ST2 (Becker et al., 2023; Langston et al., 2023). More intriguingly, a recent study showed local inflammatory signals and TCR engagement upon muscle injury drove microbiota-dependent RoRγt^+^ Tregs emanating from the gut to promote muscle regeneration (Hanna et al., 2023).

***Factors mediate Treg regenerative function in the skeletal muscle*.** Muscle injury, whether caused by acute injury or exercise, typically elicits inflammation. Not surprisingly, studies revealed that Tregs, as a critical immunosuppressive T cell subset, can control macrophage transition from inflammatory to anti-inflammatory phenotype, guarding against overexuberant production of IFN-γ by NK, CD8^+^ and conventional T cells, and consequent metabolic disruptions, particularly mitochondrial aberrancies in muscle (Langston et al., 2023). These immune-regulatory activities establish a permissive microenvironment for subsequent muscle regeneration.

Beyond these indirect effects mediated through immune cell regulation, muscle Tregs also exert direct tissue-restorative functions through production of regenerative mediators. In particular, Treg**-**derived Areg acts on MuSCs to promote their differentiation *in vitro* and *in vivo*, and also dampens expression of fibrotic effectors (Burzyn et al., 2013; Jin et al., 2018). Notably, one study in 2018 showed that administration of IL-10 and Areg, but not IL-2/anti-IL2 complexes or IL-33, can alleviate chronic skeletal muscle inflammation and damage induced by infection with Toxoplasma gondii (Jin et al., 2018), indicating the regenerative factor-producing capability of Tregs may be more effective than simply increasing their numbers.

Thus, muscle Tregs illustrate how injury-induced recruitment, IL-33/ST2-dependent accumulation, and Areg-mediated direct communication with MuSCs cooperate to promote tissue restoration (Fig. 4).

**Fig. 4:**
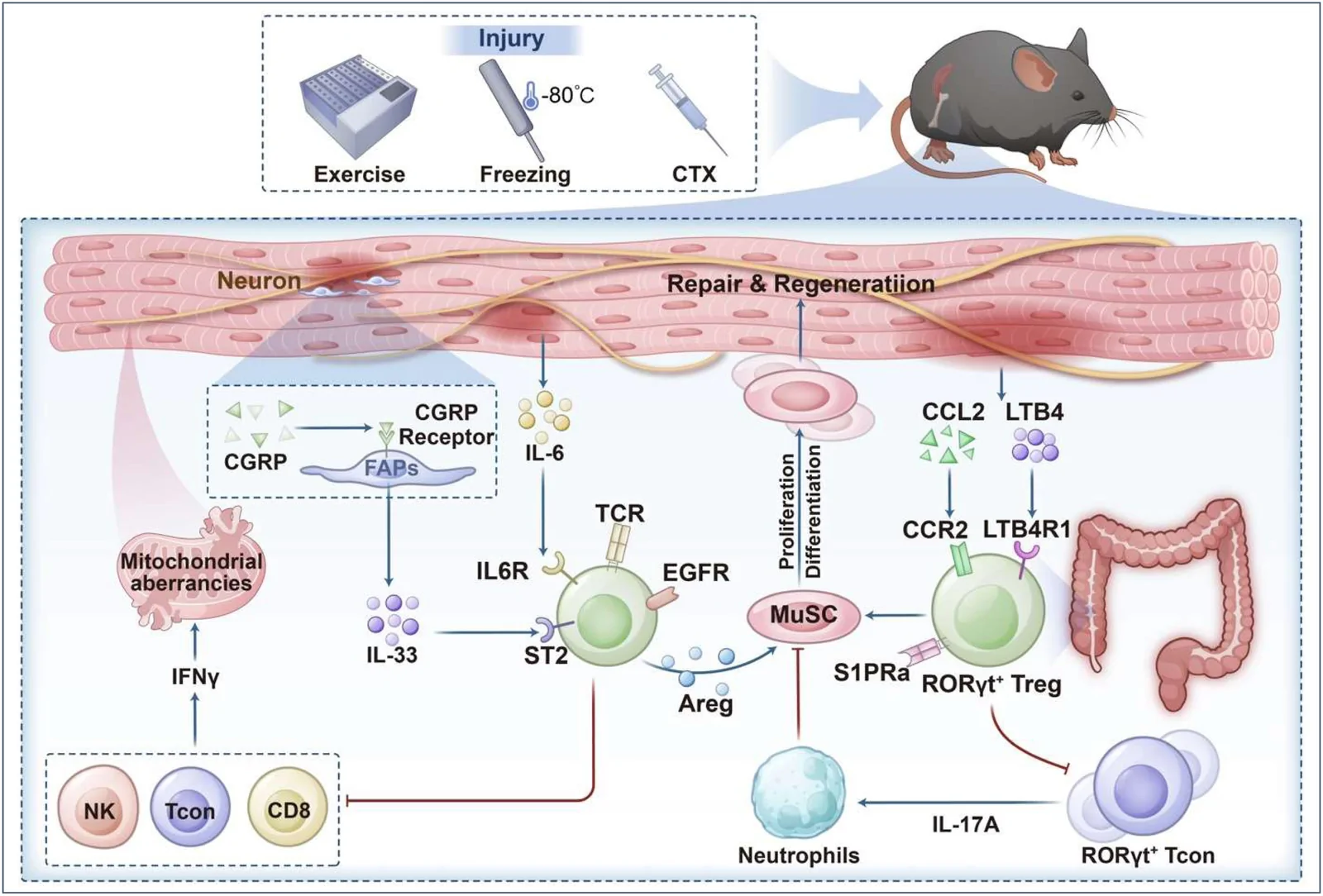
**Tregs in skeletal muscle regeneration.** Upon muscle injury, muscle sensory nerves produce CGRP, which acts on FAPs via the CGRP receptor to induce IL-33 production, thereby driving Treg accumulation through ST2. In addition, local inflammatory signals such as IL-6, CCL2, and LTB4, induced by muscle injury, drive microbiota-dependent RORγt^+^ Tregs from the colon to the muscle via CCR2 and LTB4R1. Tregs at the injured site produce Areg, which directly facilitates MuSC proliferation and differentiation, thereby promoting muscle regeneration, while keeping NK cells, conventional T cells, CD8^+^ T cells, and neutrophils in check.

### 4.2 Tregs in reparative lung regeneration

The lung acts as both the primary site of gas exchange and a barrier to the outside environment. The lung can be divided into two basic regions: the airways that conduct airflow and the distal gas-exchanging alveolar compartment. Although the lung is a highly quiescent tissue at steady state, recent studies have demonstrated their remarkable capacity to respond to injury, repairing and regenerating lost cells and tissue structure (Basil et al., 2024). The alveolar compartment consists of two major epithelial cell lineages: alveolar type 1 (AT1) and alveolar type 2 (AT2) cells, with AT2 serving as the reservoir of epithelial progenitor activity in the distal lung, capable of self-renewal and differentiation into AT1 cells after injury. Additionally, at the bronchioalveolar duct junction-the regions where the distal airways of mice empty into the alveolar compartment-a rare population of cells referred to as bronchioalveolar stem cells (BASCs) exhibits airway and alveolar epithelial multipotency in some injury scenarios (Liu et al., 2024).

Lung Tregs are infrequent, comprising 5%-10% of the CD4^+^ T cell population, and are occasionally found near bronchioalveolar duct junctions at steady state. After influenza infection or endotoxin lipopolysaccharide (LPS) challenge, Tregs expand and disperse throughout alveolar spaces as the virus spreads to distal airways (D'Alessio et al., 2009). Tregs also increase their presence after pneumonectomy (PNX), a non-inflammatory model of lung growth. Studies have shown that depletion of Tregs, in the contexts of LPS-induced lung epithelial damage, left unilateral PNX, and infection-elicited damage, led to decreased alveolar epithelial proliferation and delayed lung injury recovery (Dial et al., 2017; Mock et al., 2014). Not surprisingly, Tregs are central to suppressing lung inflammation and promote resolution through control of both innate immune cells, such as neutrophils, macrophages, and adaptive immune cells, including CD8^+^, Th1, Th2, γδT cells (Jovisic et al., 2023).

***Signals promote Treg expansion and regenerative phenotype in the lung***. It is presumed that specific signals elicited upon injury enable Tregs to transition from an inherent immunoregulatory to a regenerative mode. However, identifying such signals that can distinguish these two modalities of Tregs is quite challenging. As most studies rely on Foxp3-DTR mice to punctually yet completely deplete Tregs by short-term DT treatment, the impaired tissue regeneration observed under such strategies is likely an intertwining combination of Treg immune-regulatory and tissue-regenerative roles. However, the study of the regenerative capability of lung Tregs provides a good example to resolve such disputes. In 2015, Arpaia et al. found that deficiency of Areg production in Tregs neither affects Treg cell ratio and number, nor affects the virus load in the lung tissue compared to wild-type controls, demonstrating that Areg deficiency does not impair Treg immunosuppressive function or anti-viral immune responses in an influenza infection mouse model. Surprisingly, Treg-specific deletion of Areg indeed leads to lung tissue damage and decreased blood oxygen concentration (Arpaia et al., 2015).

Then, what signals upon injury can induce Areg expression in lung Tregs? Indeed, they found that while TCR stimulation fails to induce Areg production, the alarmins IL-33 and pro-inflammatory cytokines IL-18 not only induce the expansion of Areg-expressing Tregs, but also increase the Areg protein level on a per-cell basis (Arpaia et al., 2015). Therefore, the Treg regenerative function is mobilized by separable signals from those eliciting Treg suppressive function, the latter being stimulated by, and dependent upon, TCR signaling. Another study recently showed that in the context of severe acute respiratory syndrome coronavirus 2 (SARS-CoV-2)-induced acute lung injury (ALI), IL-6 increases Notch4 expression on lung Tregs, which, in turn, restrains production of Areg by IL-18 and promotes severe lung inflammation, thus limiting Treg regenerative functionality (Harb et al., 2021).

***Factors mediate Treg regenerative function in the lung***. In the alveolar space, AT2 cells proliferate at steady-state and regenerate lung epithelium in response to injury. Indeed, studies have shown that in the LPS-induced ALI model, CD103^+^ Tregs promote epithelial cell proliferation, suggesting that direct contact between Treg and AT2 cells is important for Treg-mediated AT2 cell proliferation (Mock et al., 2014). In a related study, Arpaia et al. showed that Treg-produced Areg promotes recovery after ALI (Arpaia et al., 2015). Mechanistically, Areg produced by Tregs induces EGFR expression on Col14^+^ mesenchymal lung cells, supporting their survival and upregulating their expression of Fgf7 and Fgf10, which in turn stimulates AT2 regeneration and facilitates ALI recovery (Kaiser et al., 2023). Consistently, human lung Tregs stimulated *ex vivo* also produce Areg and KGF (Dial et al., 2017), highlighting their potential relevance for informing clinical trials. Beyond AT2 cells, whether BASCs play a role in epithelial repair after virus infection, and whether Treg-BASC interactions are important for lung reparative regeneration awaits further study.

Thus, lung Tregs provide one of the clearest examples that selected tissue-regenerative programs can be experimentally separated from broader canonical immune-regulatory functions.

### 4.3 Tregs in physiological and reparative skin regeneration

Skin is the largest organ in our body, consisting of the epidermis, the dermis, and a bed of adipose tissue and fascia below (Kabashima et al., 2019). The skin also harbors appendages, such as hair follicles, sweat and sebaceous glands. As a physical barrier susceptible to mechanical injury, chemical, and microbial insults, its integrity is essential for survival (Belkaid et al., 2016). Skin regeneration depends on multiple stem cells located in different anatomical locations: hair follicle stem cells (HFSCs), melanocyte stem cells (MeSCs), sebaceous gland stem cells, interfollicular epidermis stem cells (IFESCs), and dermal stem cells (SSCs) (Gonzales et al., 2017). IFESCs are located at the basal layer of the epidermis and are responsible for regenerating the protective skin barrier during normal tissue renewal. HFSCs are not only responsible for regenerating hair follicles and hair shafts during natural hair growth cycle, but also can migrate and differentiate into epithelial cells to repair the epidermis during wound healing (Blanpain et al., 2006; Zhang et al., 2024).

Unlike the above-mentioned tissues such as the skeletal muscle and lung, skin harbors a large population of Tregs, consisting of 20%-60% of CD4^+^ T cells in mice, ∼20% of CD4^+^ T cells in human adult skin, compared with ∼5% in the peripheral blood and 5%-10% in human adult colon (Sanchez Rodriguez et al., 2014). It has been shown that murine skin Tregs abruptly accumulate in a defined window of postnatal day 6 to day 13, dependent on hair follicle development and commensal microbes, both of which could increase the expression of the chemokine CCL20 from hair follicles, attracting neonatal thymic Tregs expressing high levels of CCR6 (Scharschmidt et al., 2015). Additionally, a recent study reported that Tregs express CXCR4, which promotes their accumulation in the hair follicle epithelium enriched for CXCL12 (Cohen et al., 2025). As expected, skin Tregs are critical for maintaining the immune privilege of the HFSC niche (Ali & Rosenblum, 2017; Cohen et al., 2024). In 2017, the Rosenblum group first showed that Tregs fluctuate during different hair stages, and depletion of Tregs in Foxp3-DTR mice inhibits both natural hair growth and depilation-induced hair regeneration (Ali et al., 2017; Liu et al., 2022). We recently reported that such a regenerative effect of Tregs on hair growth and regeneration is mediated through glucocorticoid receptor (GR) signaling and TGF-β3 (Liu et al., 2022). Moreover, Tregs are also critical in promoting wound healing after full-thickness wounds (Lui et al., 2024; Nosbaum et al., 2016; Shime et al., 2020), and facilitating epidermal repair after epidermal abrasion by tape stripping (Luan et al., 2024; Mathur et al., 2019).

***Signals promote Treg expansion and regenerative phenotype in skin*.** Although Ali et al. reported that Treg cell numbers and proportions fluctuate during natural hair cycle progression, the signals governing these dynamics remain unknown (Ali et al., 2017). Furthermore, evidence for Treg expansion in the depilation-induced hair regeneration model is limited, with only one study showing that HFSC expressing CD80 can induce pTreg cell expansion in a tape-stripping epidermal injury model (Luan et al., 2024). Beyond numerical Treg expansion, a paramount contribution of Treg to tissue repair and regeneration is probably their intrinsic regenerative capability. Seeking the signals that trigger Treg to switch from an immune regulatory to a tissue regenerative modality, our recent study identified that GR signaling serves as a key inducer. We found that hair depilation can elevate local production of glucocorticoids in skin, but not systemically in the blood. Correspondingly, Treg-specific knockout of *Nr3c1* (encoding GR) impairs hair regeneration (Liu et al., 2022). Crucially, while glucocorticoids are broadly immunosuppressive, Treg-specific GR deletion did not undermine their immunosuppressive function across multiple inflammation models, consistent with another study showing GR signaling is not required for Treg immune regulatory function under steady state (Kim et al., 2020; Liu et al., 2022). Therefore, GR signaling preferably regulates the regenerative modality of skin Tregs without affecting their inherent immunosuppressive capacity.

The contribution of Tregs to skin regeneration extends beyond the hair follicle. For example, in a full-thickness wound model, studies showed that a marked induction of EGFR expression was detected in skin Tregs 3 days after wounding, and Treg-specific deletion of EGFR undermines wound repair. Furthermore, skin Tregs with a set of genes enriched in “wound healing” can be expanded by ultraviolet B (UVB) (Shime et al., 2020; Yamazaki et al., 2014).

***Factors mediate Treg regenerative function in skin*.** Among several different types of stem cells in the skin, the most well-studied is Treg-HFSC crosstalk. The Rosenblum group in 2017 reported that skin Tregs located around the hair follicle directly interact with HFSCs via Jag1-Notch signaling to drive HFSC proliferation and differentiation (Ali et al., 2017). Jag1 is constitutively highly enriched in skin Tregs compared to Tregs in the skin-draining lymph nodes or skin CD4^+^ conventional T cells. Moreover, the expression of Jag1 on Tregs seems to be downregulated from telogen to anagen. In addition to Jag1, our recent study further showed that TGF-β3 increased upon skin injury, can also drive HFSC activation and differentiation (Liu et al., 2022). In contrast to TGF-β1, a constitutively expressed cytokine essential for Treg immunosuppressive function, TGF-β3 expression is induced on Tregs by local GR signaling upon injury. Therefore, like Areg-expressing Treg representing a regenerative modality, TGF-β3 may similarly indicate a regenerative functionality for skin Tregs. Notably, hair regeneration is more severely delayed in GR cKO mice compared to TGF-β3 cKO mice, indicating that factors other than TGF-β3 are also involved in Treg-mediated HFSC activation. Indeed, recent studies showed that GR signaling can regulate the expression of CXCR4 on skin Tregs (Cohen et al., 2025), which promotes their attraction to and interactions with the hair follicles, or the expression of PENK, which can modulate sensory neuron activation in the skin, may directly or indirectly control hair regeneration (Mendoza et al., 2025).

Notably, in contrast to hair depilation or any other injury-induced reparative regeneration, which inevitably involves Treg immunosuppressive function, natural hair growth provides a unique model to examine physiological regeneration mediated by Tregs in the absence of overt inflammatory responses. Natural hair growth is a physiological process that operates under homeostatic conditions without triggering unwanted inflammation. Supporting this, our studies and others demonstrate that deletion of GR, Jag1, TGF-β3 in Tregs delays natural hair regeneration without inducing systemic inflammation (Ali et al., 2017; Liu et al., 2022). Therefore, these studies provide compelling evidence that certain tissue-regenerative activities of Tregs can occur without measurable impairment of their canonical immune-regulatory functions.

HFSCs are not only responsible for regenerating hair, but they can also migrate out of the niche and differentiate into keratinocytes under settings including full-thickness wounds and epidermal abrasion by tape stripping. Another study reported that Tregs promote epidermal regeneration through controlling IL-17-CXCL5-neutrophil axis of inflammation during barrier repair (Mathur et al., 2019). While such an effect of Treg seems to be dependent on their immunosuppressive functions, whether Tregs can secrete factors that directly regulate HFSC migration and differentiation into epidermal cells that repair damaged epithelium is yet to be explored. Interestingly, HFSCs themselves can also activate immune-modulatory programs during epidermal regeneration. One study reported that migrating HFSCs can acquire CD80 expression, which can induce Foxp3 expression in activated conventional T cells from circulation in the wound sites, rather than local skin-resident Tregs. These expanded Tregs can better prevent the accumulation of neutrophils (Luan et al., 2024). Therefore, such Treg-HFSC crosstalk is bi-directional during taping-induced epidermal regeneration.

In full-thickness wounds, a study from the Rosenblum group showed that skin EGFR-expressing Tregs infiltrating wounds attenuate IFN-γ-dependent accumulation of Ly6C^hi^ pro-inflammatory macrophages, and depletion of Tregs causes delayed wound closure (Nosbaum et al., 2016). Beyond such a canonical immune regulatory function, whether Tregs can produce factors to directly target epidermal cells and fibroblasts awaits exploration. Furthermore, two other studies reported UVB can induce the expansion of Tregs with a unique TCR repertoire and enriched in skin-homing receptors like CD103, CCR and P-selectin ligand and an endogenous opioid precursor, proenkephalin (PENK). Such UVB-expanded Tregs display a distinctive set of genes enriched in “wound healing-related signature”, including keratinocyte proliferation, angiogenesis, chemotaxis, and extracellular matrix, and enhance keratinocyte outgrowth through producing PENK and Areg (Mendoza et al., 2025; Shime et al., 2020; Yamazaki et al., 2014), indicating Tregs potentially can orchestrate skin repair through direct control of a variety of non-immune cells.

Thus, skin Tregs highlight the capacity of tissue-adapted Treg programs to support both physiological and injury-induced regeneration (Fig. 5).

**Fig. 5:**
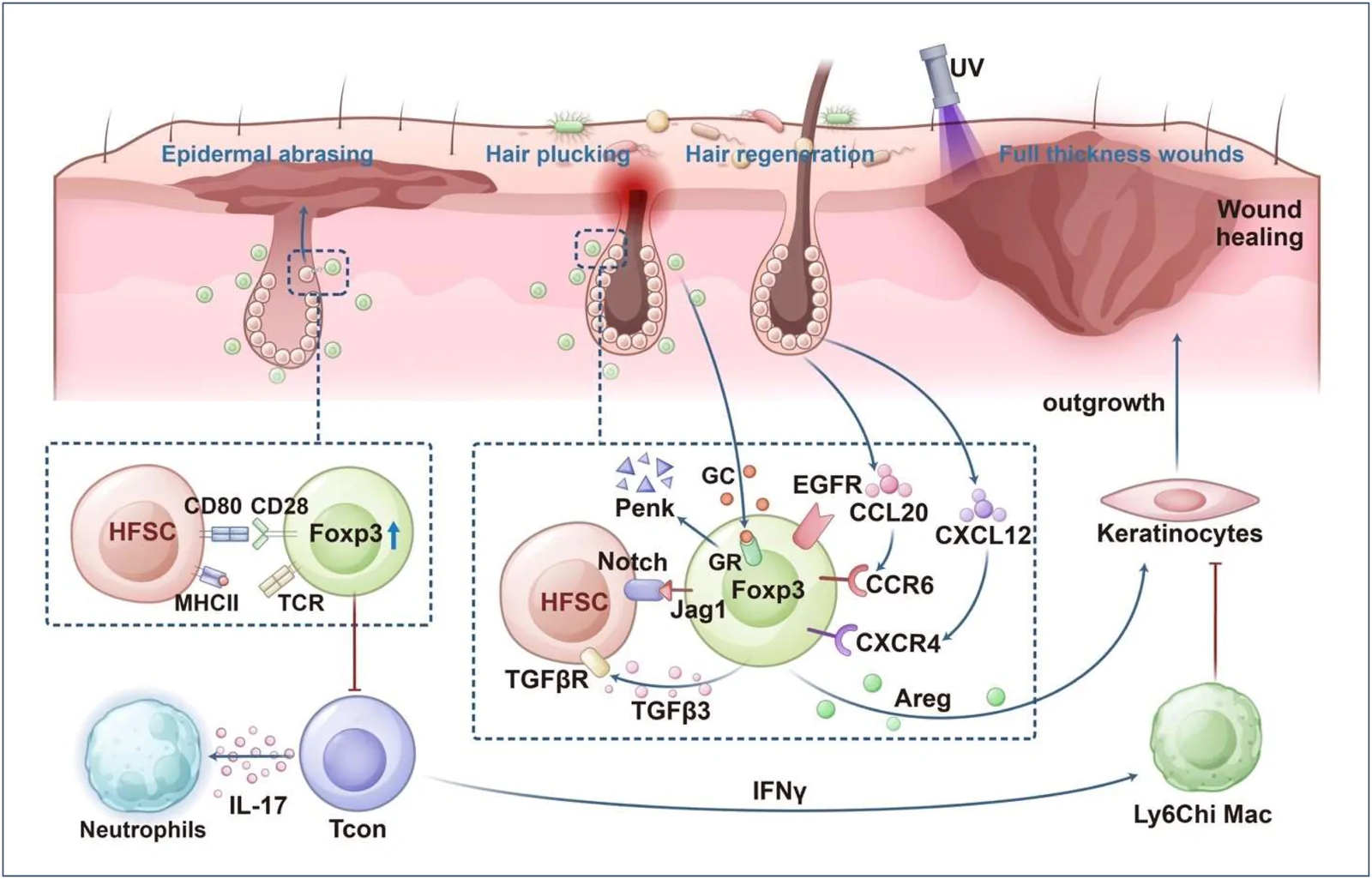
**Tregs in skin repair and regeneration.** At the steady state, Tregs are mainly localized near the hair follicle, which produces chemokines such as CCL20 and CXCL12 to attract Tregs via CCR6 and CXCR4. Following hair plucking, local glucocorticoids (GCs) trigger Tregs to produce TGF-β3, which, together with Jag1, promotes HFSC to activate, proliferate, differentiate and regenerate hair follicles. In the context of Epidermal abrasion, migrating HFSCs express CD80, which interacts with effector T cells through CD28, inducing them to upregulate Foxp3 and become induced Tregs. During full-thickness wound healing, Tregs can also produce Areg, which enhances keratinocyte outgrowth and promotes wound repair, an effect further promoted by UV-induced Treg healing programs. In addition to direct interactions between Tregs and tissue cells, Tregs suppress the activities of conventional T cells, neutrophils, and macrophages throughout the regeneration and healing process.

### 4.4 Tregs in reparative thymic regeneration

Most T lymphocytes, including Foxp3^+^ Tregs, are generated in the thymus, with thymic epithelial cell and other stromal cells providing important signals to guide a stepwise development program of different T cell lineages (Granadier et al., 2025; Liu et al., 2020; Takaba et al., 2017). The thymus is the first organ undergoing ageing and involution, and is highly susceptible to get injured by infection and irradiation, leading to reduced T cell production, making patients more susceptible to malignancies, infections, and autoimmune diseases. However, the thymus maintains regenerative capacity to deal with such insults, dependent on the repair and regrowth of thymic epithelial cells (Givony et al., 2023; Kadouri et al., 2020; Liu et al., 2020; Takahama et al., 2017). Tregs arise from two distinct progenitors: CD25^-^Foxp3^+^ progenitors and CD25^+^Foxp3^-^ progenitors, then become CD25^+^Foxp3^+^ mature Tregs. After maturation, Tregs egress into the periphery (Owen et al., 2019). Notably, Tregs can recirculate back to the thymus and suppress their de novo differentiation. New Treg cell production decreases substantially in the involuted thymus (Thiault et al., 2015). Intriguingly, a recent study by van den Brink group extends the regenerative power of Tregs to the thymus (Lemarquis et al., 2025). The authors observed that Tregs largely expand in the thymus shortly after sublethal total body irradiation (SL-TBI) or infection. By using *Rag2*^GFP^ reporter mice, they found that the enriched Treg populations in the injured thymus were mainly expanded from recirculating *Rag2*^GFP−^ Tregs, rather than newly-generated *Rag2*^GFP+^ Tregs. Furthermore, depletion and adoptive transfer of the *Rag2*^GFP−^ Treg cell population impaired and promoted thymic repair, respectively, in mice.

***Signals promote Treg expansion and regenerative phenotype in the thymus*.** Currently, little is known about the signals that can induce Treg expansion. Although thymic Tregs, like their counterparts in skeletal muscle and skin, express high levels of *Il1rl1* (encoding ST2), whether the IL-33/ST2 axis also plays a critical role in driving Treg expansion in the thymus remains unclear. *Rag2*^GFP−^ Tregs responsible for thymic regeneration highly express Areg, Tff1, and Penk. Whether there are signals upon injury that can induce the expression of these pro-regenerative factors in Tregs, or selectively promote the expansion of Treg expressing those factors, remains to be explored.

***Factors mediate Treg regenerative function in the thymus*.** Compared to *Rag2*^GFP+^ Tregs, *Rag2*^GFP−^ Tregs are enriched for genes implicated in regenerative role of Tregs: Areg, Penk and Tff1; Treg-specific deletion of Areg undermines thymic regeneration. Interestingly, *Rag2*^GFP−^ Tregs are numerically decreased and transcriptionally altered in aged mice, but adoptive transfer of young *Rag2*^GFP−^ Tregs can improve thymic regeneration. Furthermore, they also identified an analogous population of recirculating Tregs marked by ICOS^hi^ CD39^hi^ expressing high levels of Areg that exists in the human thymus. Consistently, EGFR, the receptor for Areg, is upregulated in thymic epithelial cells and fibroblasts on day 4 after injury (Lemarquis et al., 2025). Therefore, Areg/EGFR axis is critical for thymic regeneration. As for other factors such as Penk, Tff1, IL-16, and TGF-β, whether they are involved in the Treg cell-thymic stromal cell interaction remains to be explored.

Thus, thymic regeneration provides an example of injury-induced reparative regeneration in which mature peripheral Tregs acquire regenerative capacity after recruitment to a specialized tissue niche.

### 4.5 Tregs in reparative neural regeneration and remyelination

The nervous system represents a unique regenerative context because although neurons of the adult mammalian CNS generally exhibit limited regenerative capacity, axons and myelin can undergo regeneration to restore neural function (Varadarajan et al., 2022). Although Tregs are scarce in brain parenchyma at steady state, studies show that brain Tregs have a prominent regenerative role in support of myelin regeneration (remyelination) through directly signaling to oligodendrocyte progenitor cells to differentiate into myelin-producing oligodendrocytes (Liston et al., 2024).

Following experimental stroke, Tregs infiltrated the brain 1 to 5 weeks after stroke, driven by the chemokines CCL1 and CCL20 (Ito et al., 2019). In this context, Treg cell-derived osteopontin promotes microglial reparative activity through integrin receptors on microglia, thereby enhancing oligodendrogenesis and white matter repair (Shi et al., 2021). In addition, another study showed that brain Tregs can suppress neurotoxic astrogliosis through Areg-mediated inhibition of the IL-6/STAT3 pathway in microglia and astrocytes to exert their neuroprotective effects (Ito et al., 2019).

Beyond these indirect effects mediated through immune-cell regulation, Tregs can directly regulate neural progenitor populations. In a focal demyelination model induced by lysolecithin, Tregs promote oligodendrocyte progenitor differentiation and remyelination through production of CCN3 (Dombrowski et al., 2017). Similar regenerative activities have been observed in autoimmune and virus-induced demyelination models (McIntyre et al., 2020; Plaisted et al., 2016). Together, these findings identify CNS-associated Tregs as important regulators of myelin restoration through integration of immune modulation and direct communication with neural progenitor populations.

### 4.6 Tregs in cardiac reparative regeneration and remodeling

The heart is a muscular organ responsible for pumping blood through the blood vessels. Unlike the neonatal heart, which retains a regenerative capacity after birth till postnatal day 7 in the mouse, adult heart is notorious for its incapability to regenerate after injury (Li et al., 2019). Similar to the skeletal muscle, the normal heart harbors a small population of Tregs at steady state, but injury induces substantial Treg accumulation.

Studies from the Lui group in neonatal mice using apical resection found that Tregs are recruited to the injured cardiac muscle during the first week of injury, and their depletion results in more severe cardiac fibrosis. Mechanistically, Tregs directly promote proliferation of cardiomyocytes in a paracrine manner through the production of secreted factors such as CCL24, GAS6 and Areg (Hou et al., 2025; Li et al., 2019).

In adult myocardial infarction (MI), ischemia/reperfusion injury, and cryoinjury models, Tregs are similarly enriched within the myocardium. This expansion of Tregs largely results from recruitment of the circulating Treg population, whereas local proliferation also contributes to their expansion through the IL-33/ST2 axis. These expanded heart Tregs show strong expression of SPARC, which promotes collagen deposition and infarct scar formation, thereby preserving cardiac integrity after injury (Xia et al., 2020). Importantly, this example illustrates that regenerative Treg biology encompasses diverse forms of tissue restoration. In tissues with limited regenerative capacity, such as the adult heart, Tregs may primarily promote reparative remodeling and preservation of tissue function rather than replacement of lost cells.

Beyond myocardial injury, vascular Tregs contribute to tissue homeostasis in the circulatory system. During abdominal aortic aneurysm development, Tregs accumulate through recruitment from peripheral pools and produce Tff1, which regulates vascular smooth muscle cell survival (Li et al., 2022). Together, these findings demonstrate that cardiac and vascular Tregs contribute to tissue restoration through diverse mechanisms, ranging from direct support of regenerative responses to regulation of repair and remodeling processes.

### 4.7 Expanding tissue landscapes of Treg-mediated restoration

**The intestine.** The intestinal epithelium displays a remarkably high turnover rate, roughly 3 to 5 days during homeostasis, with intestinal stem cells (ISCs) fueling continuous generation of all differentiated cell types. While a large number of studies have investigated the heterogeneity of Tregs, their critical roles in maintaining intestinal homeostasis, and their interaction with microbiota (Hu et al., 2026; Ohnmacht et al., 2015; Sefik et al., 2015; Tanoue et al., 2016; Whibley et al., 2019), very few of them really look into the possible interaction between Tregs and ISCs. A recent finding by Biton et al. attempted to understand the interaction of CD4^+^ T cells and ISCs and found that pro-inflammatory cytokines, such as IFN-γ from Th1, IL-13 from Th2, promote their differentiation. In contrast, coculture with *in vitro* differentiated Tregs or the addition of IL-10, the main effector cytokine of Tregs, can enhance intestinal organoid renewal *in vitro*. Consistently, punctual depletion of Tregs in Foxp3-DTR mice leads to loss of ISCs, with an increased differentiation into mature intestinal cells, including tuft cells and goblet cells (Biton et al., 2018). Overall, a direct or even indirect role of Tregs in modulating ISCs is yet to be fully elucidated.

**The liver.** In 2025, the Arpaia group reported that Areg-producing Tregs were enriched in the livers of mice and humans with non-alcoholic steatohepatitis (NASH), a setting of chronic liver injury. Treg cell–derived Areg activated pro-fibrotic transcriptional programs in hepatic stellate cells via epidermal growth factor receptor (EGFR) signaling. Deletion of Areg in Tregs reduced NASH-induced liver fibrosis, protected mice from NASH-dependent glucose intolerance, which was also dependent on EGFR signaling in hepatic stellate cells. Areg from Tregs promoted hepatocyte gluconeogenesis through hepatocyte detection of hepatic stellate cell–derived IL-6 (Savage et al., 2024). This example further illustrates that Treg-derived mediators may have context-dependent effects, where activation of tissue remodeling pathways can contribute to either restoration or pathological fibrosis depending on disease stage and microenvironment.

**The eye.** One study in 2017 reported that in an oxygen-induced retinopathy mouse model, Tregs are transiently increased in the retina, but decline when neovascularization is established. Not surprisingly, prevention of the decline following Tregs expansion with an IL-2/anti-IL-2 mAb complex or the adoptive transfer of Tregs repairs pathological angiogenesis and the vasculature (Deliyanti et al., 2017). Another study recently showed that Tregs reside within the corneal epithelium and co-localize with limbal stem cells at steady state, and depletion of Tregs delayed corneal epithelial healing after injury (Tahvildari et al., 2024).

**The bone.** In 2024, Chen et al. found that in both human patients with bone fracture and a mouse model of bone injury, local production of CCL1 induced a massive migration of bone injury-responding CCR8^+^ Tregs from the periphery to the injury site, which in turn supported the accumulation and osteogenic differentiation of skeletal stem cells (SSCs) and thereby bone repair through secretion of progranulin (PGRN) (Chen et al., 2024) (Table 1).

**Table 1:** Summary of injury-elicited mediators and regenerative factors regulating Treg-tissue cell interaction across multiple tissues.

Tissue	Mediators influencing Treg regenerative program	Treg-producing factors regulating regeneration	Treg crosstalk within the tissue microenvironment	References
Skeletal muscle	IL-33, IL-6, CCL2, LTB4	Areg	Muscle stem cells (MuSCs)	(Burzyn et al., 2013; Kuswanto et al., 2016; Langston et al., 2023; Panduro et al., 2018; Wang et al., 2020; Cho et al., 2019; Hanna et al., 2023)
Lung	IL-18, IL-33, IL-6	Areg, KGF	Col14^+^ mesenchymal lung cells, AT2 cells	(Arpaia et al., 2015; D'Alessio et al., 2009; Mock et al., 2014; Harb et al., 2021; Kaiser et al., 2023; Dial et al., 2017)
Skin	CCL20, CXCL12, local GCs, CD80, UVB	Jag1, TGF-β3, Penk, Areg	Hair follicle stem cell, keratinocyte	(Ali et al., 2017; Liu et al., 2022; Scharschmidt et al., 2015; Cohen et al., 2025; Mathur et al., 2019; Luan et al., 2024; Shime et al., 2020; Yamazaki et al., 2014; Mendoza et al., 2025)
Thymus	IL-33?, CXCL12?	Areg, Tff1(?), Penk(?)	Thymic epithelial cell, fibroblast	(Lemarquis et al., 2025)
Central nervous system (CNS)	CCL1, CCL20, serotonin, IL-2, IL-33	Osteopontin (OPN), CCN3, Areg	Microglia, oligodendrocyte progenitor cell, microglia and astrocytes	(Shi et al., 2021; Ito et al., 2019)
Intestine	Unknown	IL-10(?)	Intestinal stem cell	(Biton et al., 2018)
Heart	IL-33	CCL24, GAS6, Areg, SPARC, Tff1	Cardiomyocyte, smooth muscle cell	(Li et al., 2019; Xia et al., 2020)
Liver	Unknown	Areg	Hepatic stellate cell	(Savage et al., 2024)
Eye	Unknown	Unknown	Limbal stem cells	(Tahvildari et al., 2024)
Bone	CCL1	Progranulin (PGRN)	Skeletal stem cells	(Chen et al., 2024)
VAT	PPAR?	OSM	Adipocyte precursors	(Wang et al., 2024)
Tendon	Unknown	Unknown	Tenocyte	(Arvind et al., 2025)
Oral mucosa	IL-33	MIF, TGF-β1	Monocyte, macrophage	(Chen et al., 2026)

## 5 General principles of regenerative Treg biology

The diverse tissue examples discussed above reveal that regenerative Treg biology represents neither a universal tissue program nor a collection of unrelated organ-specific responses, but rather a context-dependent functional state shaped by conserved and tissue-adapted mechanisms. These observations raise several fundamental questions regarding how regenerative programs are induced, regulated, and maintained. Here, we discuss overarching principles that may explain how Tregs coordinate immune regulation with tissue restoration, highlighting concepts that are supported by current evidence as well as questions that remain unresolved.

### 5.1 Do regenerative Tregs employ tissue-adapted or pan-tissue mechanisms?

A fundamental question is whether Tregs utilize common regenerative mechanisms across tissues or adopt specialized programs tailored to individual organs. Current evidence supports a model incorporating both possibilities.

On one hand, regenerative Tregs display remarkable tissue adaptation. Different tissues contain distinct regenerative niches and target cell populations, and Tregs produce specialized mediators accordingly. For example, skin Tregs utilize Jag1 and TGF-β3 to regulate HFSCs (Ali et al., 2017; Liu et al., 2022); brain Tregs secrete CCN3 to act on oligodendrocyte progenitor cells (Dombrowski et al., 2017); cardiac Tregs produce SPARC and Tff1 following myocardial injury (Xia et al., 2020) VAT Tregs produce OSM to regulate adipocyte precursors (Wang et al., 2024); and bone Tregs produce PGRN to support skeletal stem cells (Chen et al., 2024). These observations support the concept that regenerative Tregs adapt their molecular programs according to local tissue requirements.

Importantly, the biological outcomes of these tissue-adapted regenerative programs are not uniform across organs. In tissues with high regenerative capacity, regenerative Tregs may directly promote replacement of lost or damaged cellular populations, as exemplified by skeletal muscle satellite cells and hair follicle stem cells. In tissues with more limited regenerative potential, Tregs may instead facilitate reparative regeneration, remodeling, or restoration of tissue function through regulation of stromal, vascular, epithelial, or progenitor populations. Therefore, regenerative Treg biology should be viewed as a spectrum of tissue restoration processes rather than a universal mechanism of de novo tissue generation.

Despite this tissue diversity, several upstream mechanisms may provide common signals that promote regenerative Treg specialization. Among these, IL-33/ST2 signaling represents one of the best-characterized pathways promoting Treg accumulation and functional adaptation in multiple tissues. However, current evidence does not support IL-33/ST2 as a universal master regulator of regenerative Treg biology. Rather, it likely represents one component of a broader tissue-sensing network in which cytokines, alarmins, hormones, metabolic cues, and local cellular interactions collectively shape regenerative Treg responses.

On the other hand, accumulating evidence suggests the existence of conserved regenerative mechanisms shared across tissues. This possibility is particularly relevant for tissues with limited resident Treg pools at steady state, such as skeletal muscle, cardiac muscle, the CNS, eye, and bone, where injury-induced Treg accumulation largely depends on recruitment from circulating pools. Pharmacological blockade of Treg trafficking from the spleen using the S1PR antagonist FTY720 markedly reduces Treg expansion after injury in skeletal muscle and other tissues, whereas adoptive transfer of splenic Tregs promotes tissue restoration across multiple organs. Areg provides another example of a potentially conserved regenerative mechanism. Treg-derived Areg contributes to regeneration in skeletal muscle, lung, and thymus. Notably, Areg-expressing Tregs are also detected in secondary lymphoid organs, albeit at variable levels. These observations raise the possibility that circulating Areg^+^ Tregs possess basal regenerative capacity and subsequently adapt to local tissue environments after recruitment.

Collectively, these observations support a model in which, following tissue injury, a substantial number of circulating Tregs are recruited to the damaged site, rapidly adapt to the local microenvironment, undergo expansion, and upregulate tissue-adapted regenerative programs. Together with local tissue-resident Tregs, these recruited Tregs likely act through a combination of conserved regenerative factors (e.g., Areg) and tissue-specific mediators to coordinate efficient tissue regeneration.

### 5.2 Where and how do Tregs acquire regenerative functions?

An important unresolved question is whether the regenerative programs of Tregs are induced by damage signals after migration into injured tissues or are somehow pre-existing in circulating Tregs. Based on current evidence, both mechanisms may contribute.

Following the above proposed model integrating tissue-adapted and pan-tissue mechanisms, the answer appears to be context-dependent. It is plausible that a subset of Tregs, particularly those expressing shared regenerative factors like Areg, already acquire some basal regenerative capacity before they arrive at the injured site, and are primed to exert this function upon recruitment. However, even for these pre-equipped Tregs, local damage signals likely contribute to their selective expansion and further upregulation of Areg expression on a per-cell basis. Thus, local cues at the injured site are crucial for amplifying the regenerative capacity of recruited Tregs. Regarding tissue-specific regenerative programs, these are almost certainly governed by distinct, localized signals. It remains an open question whether Tregs expressing conserved regenerative factors can be subsequently instructed to upregulate tissue-specific regenerative mediators.

The signals controlling this functional transition toward enhanced tissue-regenerative activity are also incompletely understood. Unlike classical immune-regulatory functions, which are strongly influenced by TCR stimulation and antigen recognition, regenerative programs appear frequently regulated by tissue damage-derived cytokines and signals. For example, Areg expression in lung Tregs is induced by IL-18 and IL-33, but not by TCR stimulation (Arpaia et al., 2015). Likewise, TGF-β3 expression in skin Tregs is stimulated by GR signaling (Liu et al., 2022). Supporting this distinction, a study in a type I diabetes model showed that, high-affinity self-reactive Tregs preferentially express TCR-dependent Treg mediators like IL-10, TIGIT, GITR and CTLA-4, whereas low-affinity Tregs exhibited increased expression of regenerative-associated genes such as *Areg* and *Ebi3*, suggesting that the regenerative function may be influenced by signals distinct from those dominating classical TCR-driven immune-regulatory programs (Sprouse et al., 2018).

These observations raise an additional question: does acquisition of regenerative function occur at the expense of immune regulatory capacity? Current evidence suggests a complex relationship rather than a simple functional switch. At the population level, evidence supports temporal coordination between immune regulation and regeneration. Time course profiling of Tregs by bulk RNA-seq after thymic injury revealed a rapid upregulation of Treg immune suppressive genes (e.g., *Ctla4*, *Ikzf2*, *Ikzf4*, *Tnfrsf4*) by day 1 post-injury, which then declined, while the regenerative program marked by *Areg* expression gradually peaked by day 7 post-injury (Lemarquis et al., 2025). However, whether this functional transition occurs within individual cells remains unresolved. Single-cell analysis of lung Areg^+^ Tregs identified distinct populations expressing either predominantly immune-regulatory genes or combined immune-regulatory and tissue-regenerative genes (Loffredo et al., 2025). Whether these represent different functional states of the same lineage or stable subpopulations remains an open question.

### 5.3 Does a distinct, stable regenerative Treg subset exist?

Since the discovery of regenerative functions of Tregs, a central question has been whether these functions are mediated by a dedicated regenerative Treg subset or represent a transient state adopted by conventional Tregs in response to tissue injury. Addressing this question requires consideration of several criteria. A specialized regenerative Treg subset would ideally possess: (1) robust regenerative activity that can be experimentally distinguished from generalized immune regulation; (2) definable molecular features (e.g., a unique transcriptional signature and/or surface phenotype), enabling identification and isolation; and (3) functional stability, maintaining regenerative programs even after resolution of injury-associated signals.

Currently, no study has definitively satisfied all three criteria. Nevertheless, emerging single-cell RNA transcriptomic studies provide evidence supporting the existence of regenerative Treg states. Given the central role of Areg in regeneration across multiple tissues (muscle, lung, thymus, skin), Areg-expressing Tregs may represent a separate stable regenerative subtype from the immunosuppression-oriented Treg subset. Supporting this, scRNA-seq profiling of Areg^+^ Tregs in mouse models of lung infection and fibrosis revealed a CCR8^+^Areg^+^ Treg population that preferentially expressed a “tissue repair” gene module (Loffredo et al., 2025). During thymic regeneration, scRNA-seq analysis identified a Treg subset highly enriched for regenerative factors (Areg, Tff1, Penk), distinct from other subsets focused on immunosuppressive genes (Lemarquis et al., 2025). These observations suggest that a specialized regenerative population may exist.

However, whether these populations represent a stable lineage or a transient injury-induced functional state remains unclear. Although alarmins such as IL-33 and IL-18 can induce Areg expression at the population level, it remains unknown whether these signals establish a dedicated regenerative Treg identity. Likewise, whether tissue-specific regenerative mediators such as Jag1 and TGF-β3 are expressed by a shared regenerative Treg population or by distinct tissue-resident Treg sublineages remains unresolved.

A related but fundamental question concerns the developmental origin of regenerative Tregs. Unlike canonical Treg identity, which is strongly associated with Foxp3-dependent lineage specification, regenerative capacity does not appear to be restricted to a single developmental pathway. Both tTregs and pTregs, as well as resident and recruited Tregs, can contribute to tissue-regenerative responses depending on context. For example, skin Tregs are predominantly tTregs at steady state and support natural hair regeneration, whereas pTregs contribute to repair responses after epithelial injury (Ali et al., 2017; Liu et al., 2022; Luan et al., 2024). Similarly, microbiota-induced RORγt^+^ pTregs arising in the colon have been implicated in skeletal muscle regeneration (Hanna et al., 2023). Whether developmental origin influences the propensity of Tregs to acquire regenerative programs remains an important unresolved question.

The metabolic requirements underlying regenerative Treg states represent another emerging area of investigation (Harm et al., 2025; Shi et al., 2019). Compared with conventional effector T cells, Tregs exhibit distinct metabolic adaptations that support their survival, persistence, and suppressive functions, including preferential engagement of oxidative phosphorylation and lipid metabolism under specific contexts (Michalek et al., 2011). Moreover, tissue-adapted Tregs acquire specialized metabolic programs that contribute to their functional identity, exemplified by PPARγ-dependent lipid metabolic adaptation in VAT Tregs (Cipolletta et al., 2012). Nutrient-sensing pathways, including mTOR signaling, further integrate environmental cues with Treg activation and specialization (Zeng et al., 2013). Whether regenerative Tregs require additional metabolic remodeling beyond conventional tissue adaptation remains unknown. Acquisition of regenerative functions involves coordinated migration, local expansion, production of tissue-modulating mediators, and adaptation to injury-associated microenvironments, all of which may require substantial metabolic flexibility. Determining whether metabolic reprogramming actively drives regenerative Treg specialization or instead represents an adaptation that supports tissue-specific regenerative functions will be an important direction for future investigation.

Similarly, the molecular mechanisms that establish and stabilize regenerative programs remain poorly defined. Unlike Foxp3, which characterizes Treg lineage identity, no single master transcription factor or defining epigenetic signature currently defines regenerative Tregs. Instead, regenerative states likely emerge through the integration of lineage-associated factors, tissue-adaptation pathways, and local environmental signals. For example, our recent study in skin Tregs demonstrated that GR cooperates with Foxp3 to regulate TGF-β3 expression, a key mediator of hair regeneration, illustrating how tissue-derived signals can be integrated with the core Treg transcriptional network to establish regenerative programs (Liu et al., 2022). Beyond individual transcriptional regulators, epigenetic mechanisms that stabilize tissue-adapted Treg identities, including enhancer remodeling and changes in chromatin accessibility, may provide a molecular basis for maintaining regenerative competence. Defining how transcription factors, epigenetic landscapes, and tissue-derived signals cooperate to establish durable regenerative Treg states represents an important direction for future investigation.

Therefore, regenerative Tregs are currently best considered functional states rather than established lineages. Resolving whether regenerative capacity represents a transient adaptation or a stable cellular identity will require lineage tracing, spatially resolved multi-omics, metabolic profiling, and functional validation.

### 5.4 To what extent do regenerative Treg functions contribute to physiology and disease?

While most studies have examined regenerative Treg functions following injury, emerging evidence suggests that these programs also contribute to physiological tissue maintenance. Natural hair follicle regeneration provides one example occurring under homeostatic conditions. Deletion of GR, Jag1, TGF-β3 specifically in Tregs delays physiological hair regeneration without inducing systemic inflammation (Ali et al., 2017; Liu et al., 2022). Similarly, VAT Tregs, initially recognized for regulating insulin sensitivity through suppression of VAT inflammation (Bapat et al., 2015; Feuerer et al., 2009; Li et al., 2018b, 2021), also modulate steady-state metabolic homeostasis by producing OSM-mediated regulation of adipocyte precursors (Wang et al., 2024). In the bone marrow, specialized CD150^+^ Tregs within the hematopoietic stem cell (HSC) niche preserve HSC quiescence and immune privilege through adenosine-mediated mechanisms (Hirata et al., 2018). Together, these findings indicate that regenerative Treg functions contribute not only to injury responses but also to normal tissue maintenance.

The clinical implications of impaired regenerative Treg function remain largely unexplored. While defective immune-regulatory activity of Tregs is well-established in autoimmune diseases, it remains unclear whether impaired regenerative programs contribute to human disorders characterized by defective tissue restoration, including impaired wound healing, ageing-associated decline, sarcopenia, or metabolic dysfunction.

Pregnancy represents a particularly intriguing context in which Treg functions may extend beyond immune tolerance. Tregs are indispensable for maintaining maternal-fetal immune tolerance, and their dysfunction is associated with adverse outcomes including fetal resorption, recurrent miscarriage, endometriosis and preeclampsia. Whether these complications also involve defective Treg regenerative support of placental or fetal development remains largely unexplored. Supporting this possibility, the accumulation of memory Tregs in the second pregnancy is associated with improved fetal growth (Thiele et al., 2025). Disentangling immune regulatory and regenerative contributions in pregnancy represents an important future research direction. A major challenge in defining the clinical relevance of regenerative Tregs is the substantial functional redundancy among Tregs and other immune or stromal populations, which may compensate for or mask Treg-specific contributions. Ageing provides another promising context in which to investigate long-term consequences of altered regenerative capacity. Whether deficiencies in the regenerative Treg programs contribute to human ageing-related disorders in tissue maintenance, including sarcopenia, delayed wound healing, or metabolic decline, remains an important question for future studies.

## 6 Harnessing Tregs in regenerative medicine

Treg cell therapies are currently being evaluated in clinical trials for autoimmune diseases, allergic disorders, and prevention of transplant rejection, largely by exploiting their well-established immunoregulatory functions (Ferreira et al., 2019; Raffin et al., 2020; Wardell et al., 2025). In contrast, despite growing evidence that Tregs directly promote tissue homeostasis, repair, and regeneration across diverse organs, relatively few therapeutic strategies have been specifically designed to harness their regenerative potential. Nevertheless, recent preclinical studies in mouse models have yielded encouraging results and can be broadly classified into four complementary approaches: (1) expansion of endogenous Tregs, (2) adoptive transfer of polyclonal Tregs, (3) tissue- or antigen-specific engineered Tregs, and (4) direct delivery of Treg-derived regenerative mediators.

**Expansion of endogenous Tregs**. The simplest strategy is to expand endogenous Tregs using cytokines or other regenerative cues. Low-dose IL-2, IL-2/anti-IL-2 complexes, IL-33, and related approaches efficiently increase Treg numbers *in vivo* and have shown encouraging efficacy in multiple preclinical models. For example, intramuscular IL-33 administration enhances Treg accumulation and improves skeletal muscle regeneration after injury (Kuswanto et al., 2016). However, simply increasing Treg numbers is not always sufficient. In the aged central nervous system, expansion of Tregs using IL-2/anti-IL-2 complexes fails to restore oligodendrocyte differentiation or remyelination because aged Tregs exhibit intrinsically impaired regenerative capacity (de la Fuente et al., 2024). These findings suggest that successful regenerative therapy depends not only on the quantity of Tregs but also on their ability to acquire or maintain regenerative programs.

**Adoptive transfer of polyclonal Tregs**. Adoptive transfer of *ex* *vivo*-expanded polyclonal Tregs represents the most mature cellular therapy currently entering clinical practice. In preclinical studies, transferred splenic Tregs enhance regeneration in injured muscle, bone, skin, and thymus through both classical immunoregulatory mechanisms and direct interactions with tissue-resident cells (Lemarquis et al., 2025; Raffin et al., 2020). Although tissue Tregs display organ-specific transcriptional programs, these findings suggest that circulating Tregs retain considerable plasticity. Upon entering injured tissues, transferred Tregs may rapidly adapt to local microenvironmental signals, establish tissue residency, and activate both shared regenerative programs, such as Areg production, and tissue-specific reparative pathways. Consistent with this concept, recent studies indicate that, with the exception of the intestine, Tregs in most non-lymphoid organs predominantly arise from broadly self-reactive Tregs capable of transient multi-tissue migration before acquiring tissue-specific identities (Hou et al., 2025).

**Tissue- or antigen-specific engineered Tregs**. Recent advances in cellular engineering provide opportunities to optimize Treg regenerative therapies. TCR-engineered Tregs and CAR-Tregs can selectively accumulate within target tissues or recognize tissue-specific antigens, thereby increasing therapeutic precision while minimizing systemic immunosuppression (Zou et al., 2026). Although these approaches are currently being developed primarily for transplantation and autoimmune diseases, the same principles could be extended to regenerative medicine by directing Tregs toward injured organs or biomaterials. Furthermore, engineering Tregs to overexpress regenerative mediators such as Areg, Jag1, TGF-β3, and other tissue-specific reparative molecules may further enhance their regenerative efficacy.

**Direct delivery of Treg-derived regenerative mediators**. As increasing numbers of regenerative mediators are identified, direct delivery of Treg-derived molecules may represent an alternative therapeutic strategy that bypasses cell therapy altogether. Recombinant proteins, gene therapy, mRNA delivery, extracellular vesicles, or biomaterial-based local release systems could potentially reproduce selected regenerative functions of Tregs while avoiding challenges associated with cell manufacturing and persistence. However, because Tregs coordinate multiple regenerative pathways simultaneously and dynamically respond to tissue injury, individual mediators are unlikely to fully recapitulate the complexity of Treg biology.

Collectively, these strategies suggest that regenerative Treg therapy will likely evolve from simply expanding Treg numbers toward selectively enhancing their regenerative functionality. Future therapies may combine tissue-targeting technologies with engineering approaches that reinforce regenerative programs while preserving canonical immunoregulatory activity, rather than simply increasing Treg abundance. A deeper understanding of the molecular mechanisms governing the transition from immunoregulatory to regenerative Treg states will be essential for developing next-generation Treg therapies capable of promoting tissue repair and regeneration across diverse clinical settings.

## 7 Concluding remarks

The expanding investigation of Tregs in the non-lymphoid tissues has challenged the long-held paradigm of these cells as dedicated immune suppressors. Beyond their established role in maintaining immune tolerance, Tregs have emerged as active coordinators of tissue homeostasis, physiological regeneration, and restoration following injury across diverse organs. Through specialized tissue-regenerative programs, Tregs directly communicate with stem cells, stromal cells, epithelial cells, and other parenchymal populations to regulate physiological renewal, reparative regeneration, and restoration of tissue function after injury.

Notably, the emerging dialogue between Tregs and non-immune tissue cells is far more extensive than previously appreciated. For instance, acute and punctual depletion of Tregs induces more profound and rapid changes in non-immune cells like fibroblasts, endothelial cells, than traditionally viewed immune cells like APCs and effector T cells (Glasner et al., 2023). Furthermore, the regenerative function may represent evolutionarily conserved properties of Tregs, as illustrated by a study showing that Foxp3^+^ T cells promote the regeneration of spinal cord, heart, and retina in zebrafish (Hui et al., 2017).

We thus propose that ‘Treg’ designates a uniquely versatile T cell lineage equipped with two complementary functional modalities: a conserved immune-regulatory program that controls immune response and a tissue-regenerative program that enables direct coordination of tissue maintenance, repair and regeneration. These programs are mechanistically distinguishable in selected experimental settings, yet functionally integrated during physiological responses to tissue stress and injury. Thus, regenerative Treg biology does not represent a replacement of classical immune regulation, but rather an expanded functional repertoire that allows Tregs to serve as both guardians of immune homeostasis and orchestrators of tissue restoration.

Important questions remain regarding how regenerative Treg programs are induced, stabilized, and adapted across tissues. Defining the developmental origin, metabolic requirements, and molecular regulators of regenerative Treg states will not only refine our understanding of Treg biology but may reveal new therapeutic opportunities. Current Treg-based strategies, largely focused on autoimmunity, transplantation, and cancer, may ultimately be expanded toward promoting tissue repair, regeneration, and healthy ageing.

## CRediT authorship contribution statement

**Yannan You:** Writing – original draft. **Xianting Hu:** Writing – original draft. **Yueqing Xue:** Writing – original draft. **Zhi Liu:** Writing – review & editing, Writing – original draft, Supervision, Investigation, Funding acquisition, Conceptualization.

## Declaration of competing interests

The authors declare that they have no known competing financial interests or personal relationships that could have appeared to influence the work reported in this paper.
